# Does Internet voting make elections less social? Group voting patterns in Estonian e-voting log files (2013–2015)

**DOI:** 10.1371/journal.pone.0177864

**Published:** 2017-05-18

**Authors:** Taavi Unt, Mihkel Solvak, Kristjan Vassil

**Affiliations:** Johan Skytte Institute of Political Studies, University of Tartu, Tartu, Estonia; Universite Toulouse 1 Capitole, FRANCE

## Abstract

Remote Internet voting places the control and secrecy of the immediate voting environment on the shoulder of the individual voter but it also turns voting into yet another on-line activity thus endangering the well-known social nature of voting and possibly reducing the crucial sense of civic duty that is important for a healthy democracy. There is however a complete lack of evidence to what degree this actually materializes once electronic voting is introduced. This paper uses individual level log data on Internet voting in Estonian elections between 2013–2015 to inspect if Internet voting retains the social nature of the voting act. We do so by examining if Internet voting in groups takes place and what implications it has for voting speed. We find strong evidence of e-voting in pairs. Same aged male-female pairs seem to be voting in close proximity to each other, consistent with spouses or partners voting together. Also, female-female and female-male pairs with large age differences seem to be voting together, consistent with a parent voting with an adult aged offspring. With regards to voting speed we see the second vote in a vote pair being considerably faster than the first vote, again indicating a shared voting act. We end with a discussion of how the onset of electronic voting does not make elections less social, but does make vote secrecy more a choice rather than a requirement.

## Introduction

Voting in elections remotely over the Internet is arguably the future mode of political participation [1]. It makes the trip to the polling station redundant and will bring unprecedented agility to the voting process, including considerable time-saving for citizens. Yet it might also change the nature of how we vote in two important aspects.

The first is the relaxation of control of the immediate voting environment and placing the responsibility of vote secrecy partially on the client side. It is up to the voter to choose a secure and secret “virtual polling booth” in the form of an Internet connected device. The same applies to keeping the decision process to oneself and making the final vote choice alone. The election administrator can only provide a secure voting solution, but not control the environment in which the client chooses to implement the solution or the particular manner how the person votes.

The second, and arguably a more fundamental change, is reducing voting to an individual on-line activity and thereby causing elections to become less social. If the voting ritual is reduced to a point and click exercise on par with other more trifle things people do on-line, then the social responsibility and a feeling of civic duty that should drive the vote decision is reduced, thereby undercutting a crucial element for a well-functioning democracy.

These concerns arise out what we know about electoral behavior so far. More than half a century of electoral research has shown the social environment to take a heavy influence on the voting act. Starting in the 1950s various election studies have demonstrated that people share their political decisions, discuss them among friends and family and frequently vote together, i.e. they act in unison save for entering the voting booth in pairs. This phenomenon has also been demonstrated to drive turnout, meaning that a voter in a family increases the participation probabilities of other family members. Internet voting could reduce this communal aspect of voting by increasing the social isolation of the voter. We do not really know, however, if these fears are justified or overblown due to the small number of countries currently offering remote Internet voting and the nature of the data needed to see evidence for it.

We aim to remedy this lack of knowledge by examining to what degree are group voting patterns visible in individual level log data on e-voting sessions and whether it has implications for how these sessions play out. Next we give a brief overview of internet voting in Estonia and proceed to discuss first to what degree has the social nature of voting in elections been examined in the past and based on that state our expectations. This is followed by an overview of the data and operationalization of the concept of group voting. Finally, we provide empirical evidence for the existence of group voting and conclude with a discussion.

## Internet voting in Estonia

Estonia is a small nation state in Northern-Europe with a population of 1.3 million people. The country regained independence in 1991 and is a member of the European Union and NATO as of 2004.

Estonia is the first country in the world to offer unlimited nationwide remote Internet voting. The Internet voting system emulates the double-envelope logic of postal voting. Voters use their electronic IDs, either by way of a smart card or a cellphone with a public key infrastructure capable SIM-card, to authenticate themselves. Voting itself happens through an e-voting client application where after authentication the candidate list is downloaded and the voter can select the preferred candidate and digitally sign the vote. The inner envelope with the vote is encrypted by the client application and the outer envelope is digitally signed by the voter to be cast over the Internet to the vote receiving server. The process itself is very fast with median Internet voting speed being between 1:21 to 1:36 minutes in 2013–2015.

Remote electronic voting has been available since 2005 and in the latest national election in 2015 every third vote (30.5%) was an e-vote, i.e. cast remotely over the Internet. As of now eight elections with Internet voting have been conducted and a total of more than 750 000 valid Internet votes have been cast. For a more technical description of the technology of electronic voting in Estonia see Heiberg *et al* [2, 3] and for a detailed analysis of usage patterns over 10 years see Solvak and Vassil [4].

## Social nature of voting

### Theory

Classical accounts see the voting act as a primarily social act, aptly summarized by Lazarsfeld’s et al. “/…/ a person thinks politically as he is socially” [5]. Accordingly, voting tends to be seen as driven by the social context the voter finds herself in [6]. This context encompasses the narrower and wider group or social network membership of the individual. The mechanism of how context defining groups influence the behavior of individual members is either through instilling shared attitudes that drive the given behavior in the group or a simple desire to conform to dominant group behavior, i.e. to be accepted as a typical member of the group.

Empirical evidence in support of context driven individual political behavior is plentiful. Lazarfeld et al. found indications that “people vote ‘in groups’” ([5], p.137), i.e. they vote like their family and friends vote. Families, be it spouses, offspring or parents, tend to turn out in elections together and the presence of one voter in a household in itself has been shown to increase the participation likelihood of other family members in itself [7–11]. This applies also specifically to young adults, who are more likely to vote when their parents do so [6, 11–13]. It is furthermore worth pointing out that the family effects tend to be stronger than those of other social networks, such as neighborhoods [11], explainable by the intensity of communications in this primary group relative to others [14]. These very same associations have been observed to hold true for the party choice/loyalty in very different societies at various time points—the narrower and wider social context influences not only the turnout decision, but also individual vote choice [15–17]. People discuss their political preferences within these social networks and this in turn shapes their individual choices [16, 18].

Largely absent from the literature is the implications of group voting on the nature of the physical voting session as such. If the vote is indeed a group vote then it should lead to a simplification of the voting process in the sense that vote relevant information has been discussed and the final decision making should be more resolute as a result. Furthermore, even if vote relevant information is not really discussed in the group and only the vote choice is shared, it should still have an impact given that the decision of a group member is a cognitive informational shortcut for other members. Close knit groups, such as families or spouses, frequently employ information shortcuts, assuming decisions by others proceed from a shared interest of the group.

Internet voting might change this social aspect of voting. There is plentiful evidence that on-line activity can lead to social isolation of individuals [19–21] Applied to elections, a move to on-line cancels the need for a shared trip to the polling station in families and removes the otherwise ceremonial nature of the exercise [22]. The voting act can become fully private, reducing further the social pressure to act in a common interest, which would be there with paper based elections and with a norm to participate in the community [23]. It also reduces potential exposure to political discussions while engaging in the collective exercise of democratic participation.

To sum up, evidence clearly points towards a shared voting experience. People take cues from the behavior of people close to them and engage in voting together. They also share their political preferences in closer circles and discuss their choices and this has positive implications for turnout given that a voter in a household makes voting by other family members also more likely. We point out that these practices stand in marked contrast to the formal requirement that the vote should be an individual decision executed in an environment that ensures secrecy. A move to on-line elections, however, might reduce the shared nature of the voting act and indeed make it a more private exercise by getting rid of the ritual of voting in a polling station together with other citizens on the Election Day.

### Research question

The voting studies cited above hold for paper based voting and have been established using various survey methods. The fear is that the advent and spread of remote Internet voting has the potential to make the “companion effect” ([11], p.856) in voting less pronounced. However, given that absence of evidence is not evidence of absence, we have to flip the perspective and look for positive evidence for the social nature of Internet voting. The research question is therefore simple and straightforward:

*Does Internet voting exemplify patterns of group voting*?

If we find distinct non-random patterns indicative of group voting, we can dismiss the potential danger to the social nature of elections, if not, we have to conclude that established evidence for paper based voting is not mirrored in case of Internet voting and there is cause for concern about the reduction of civic values in times of Internet voting. The discussion above also points towards what kind of patterns would be likely in the data if Internet voting does not make elections less social. If the companion effect holds and spouses or partners e-vote together we should see pairs of roughly same aged voters with differing genders e-voting together. If the effect of parent vote on the vote by young adults holds we should see pairs of voters with large age differences e-voting together. Finally, if the information on the vote is indeed shared, then we should see associations with the speed of the voting process as the vote by one acts as a cognitive shortcut for others. We assume therefore group votes to be cast faster when compared to votes cast by lone voters.

The next section explains what kind of data we utilize to study the problem and how do we define group voting.

## Data and definitions

Individual level voter turnout studies using surveys rely on self-reported behavior, which suffers from non-random recollection and in the case of turnout also from over-reporting [24].

We have the luxury of employing actual data on behavior in the form of system log data of Estonian Internet voting in 2013, 2014 and 2015, comprising close to 414 000 remote Internet voting sessions. The logs were recorded during e-voting for system monitoring purposes and were later cleaned and anonymized for scientific study. The logs include the voter gender, age, anonymized IP, operating system type used on the voting computer and two timestamps, one when the voter received the candidate list in the voting app after authenticating herself and a second one when the vote was cast. We have of course no information on the votes themselves, these were encrypted by the voting application, separated from the personal information before vote counting and then destroyed after the election. We are only operating with limited anonymized data on the outer envelopes of the encrypted votes.

The anonymized IP allows to see how many e-votes were cast with the same IP address, it is essentially a grouping variable for the voting sessions. How many votes were cast using shared IPs is shown in Table 1. We see that the largest group is only one voter per IP. Two voters using the same IP is also quite frequent, it occurred 25 266 times in 2015, for example. Three voters per IP is already less common, only 5902 such occurrences were observed in 2015. We see that there are also some cases of more than 100 voters per IP, but this might be due to a large set of computers sharing an IP in big companies or institutions where people happen to e-vote during working hours.

**Table 1:** Number of voters per IP in 2013, 2014 and 2015.

Number of voters per IP	2013	2014	2015
1	40 625	31 966	46 795
2	19 488	13 641	25 266
3	4 243	2 951	5 902
4	1 577	1 100	1 937
5	670	553	703
6	395	399	380
7	252	306	256
8	176	227	211
9	143	191	182
10	124	183	177
11–15	308	411	634
16–20	140	98	374
21–50	298	114	544
51–100	37	29	42
> 100	27	22	28

We now face the question of how to define group voting. Even though we have very precise indicators for voting sessions in the form of log data we do not actually know how voters proceeded to vote, if, for example, they voted in the same room with others present or not.

**Fig 1:**
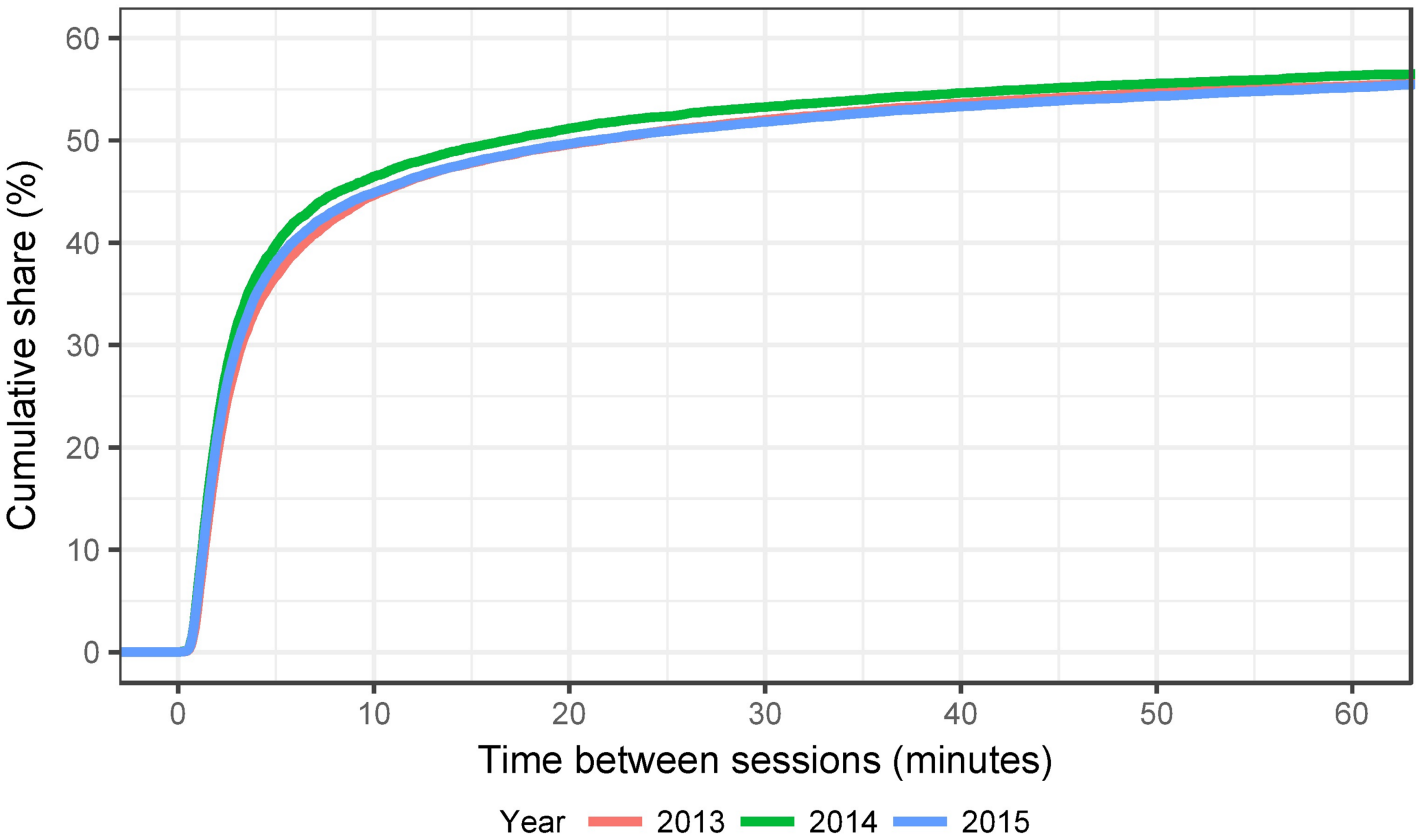
Cumulative distribution of time between e-voting sessions with shared IPs.

We have to employ certain heuristics to arrive at a definition that should capture the most frequent instance of a shared voting experience. First we focus in detail on IPs with two individual voters. We also use only voters who voted once. The Estonian system allows to e-vote multiple times with the last one counting, but 97–98% of e-voters vote only once ([4], p.82). In addition, we drop separate IP sharing voting sessions that are overlapping in time, as well as sessions that use different operating systems. Both instances would indicate that separate devices were used by voters to vote. Using only session pairs with the same operating system of course does not necessarily mean that the same computer was used, but it does reduce the possibility of separate devices being used in the voting process. We also drop sessions that were longer than 30 minutes and had more than 10 minutes between the end of the first and the beginning of the second session. This should ensure that we are indeed observing shared voting sessions and Fig 1 shows that close to 45% of all voters sharing an IP indeed vote within 10 minutes of each other.

Finally, we select only sessions that happened during the weekend or between 6 pm to 12 pm during weekdays to reduce the possibility of observing work colleague pairs and increase the probability of observing pairs that form a household. To sum up, our definition of a group vote is the following:

*exactly two separate voters using the same IP*, *a computer with the same operating system*, *voting within 10 minutes of each other between 6 pm to 12 pm or during the weekend*.

We employ these strict limitations to get as close to a likely family voting situations as possible. Ideally, these restrictions would capture situations where members of a family or household vote in close proximity to each other using the same computer—something that would be a “companion effect” in case of Internet voting.

## Results

Table 2 shows how many session pairs match the group vote definition, depending on the election it is 6.9 to 7.9 percent of all e-voters.

**Table 2:** Paired e-voting sessions.

Year	IP pairs	Number of voters	Share out of all e-voters (%)
2013	5 237	10 474	7.9
2014	3 607	7 214	7.0
2015	6 093	12 186	6.9

The relatively large number of exactly two votes per IP has been noticed before, Heiberg et. al point out that 1.95, 1.97 and 2.11 persons on average shared and IP in 2013, 2014 and 2015 elections respectively ([3], p.34). They also noticed that the distribution of these votes was not even over the voting period with people sharing and IP tending to either vote in temporally overlapping sessions or in close temporal proximity to each other ([3], p.13).

### Age patterns

**Fig 2:**
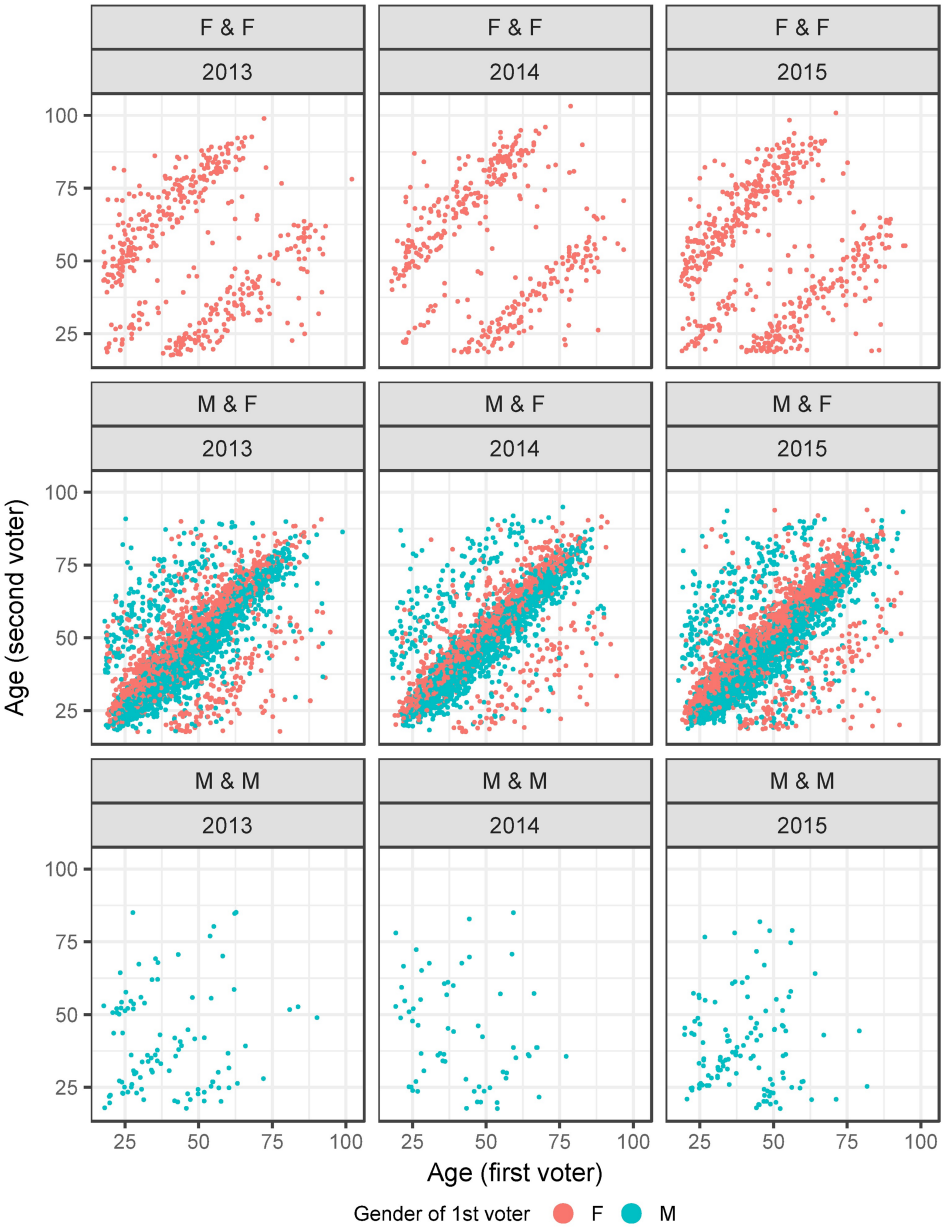
Age patterns for voter pairs (female-female, male-female and male-male pairs).

The age patterns for paired e-voters are pictured in Fig 2, we show three sets of scatterplots where the age of the first voter is on the x-axis and age of the second voter on the y-axis. The first row of scatterplots shows female-female, the second male-female and the third row male-male voter pairs; the gender of the first voter is also color marked.

The first row shows two distinct higher density areas on the scatterplot, one below the diagonal and one above it. This means we observe female-female pairs where either the first voter is by a wide margin younger than the second voter (high density area above the diagonal) or the other way around (high density below the diagonal). We do not, however, observe many cases of same aged female-female pairs (not much density on the diagonal). The patterns are therefore consistent with a mother-daughter pair voting together.

The second row showing male-female pairs is also distinct in its patterns. The highest density is along the diagonal, meaning same aged male-female voters e-voting together, which is consistent with spouses or partners voting in unison. Two distinct high density areas above and below the diagonal are also visible marking male-female pairs where one voter is clearly older or younger than the other. We also point out that between the high density area on the diagonal and the ones above and below it, are less populated empty areas, this means the age differences for the pairs off the diagonal are clearly quite large, which would be the case when father-daughter or mother-son pairs are voting together. In total we observe slightly more “male first, female second” voter pairs, those make up 58% of all voter pairs with gender difference.

The scatterplots for male-male pairs on the third row show lower density patterns, with only the year 2013 indicating a clearly similar picture as in the first row for female-female pairs.

### Patterns of session length

**Fig 3:**
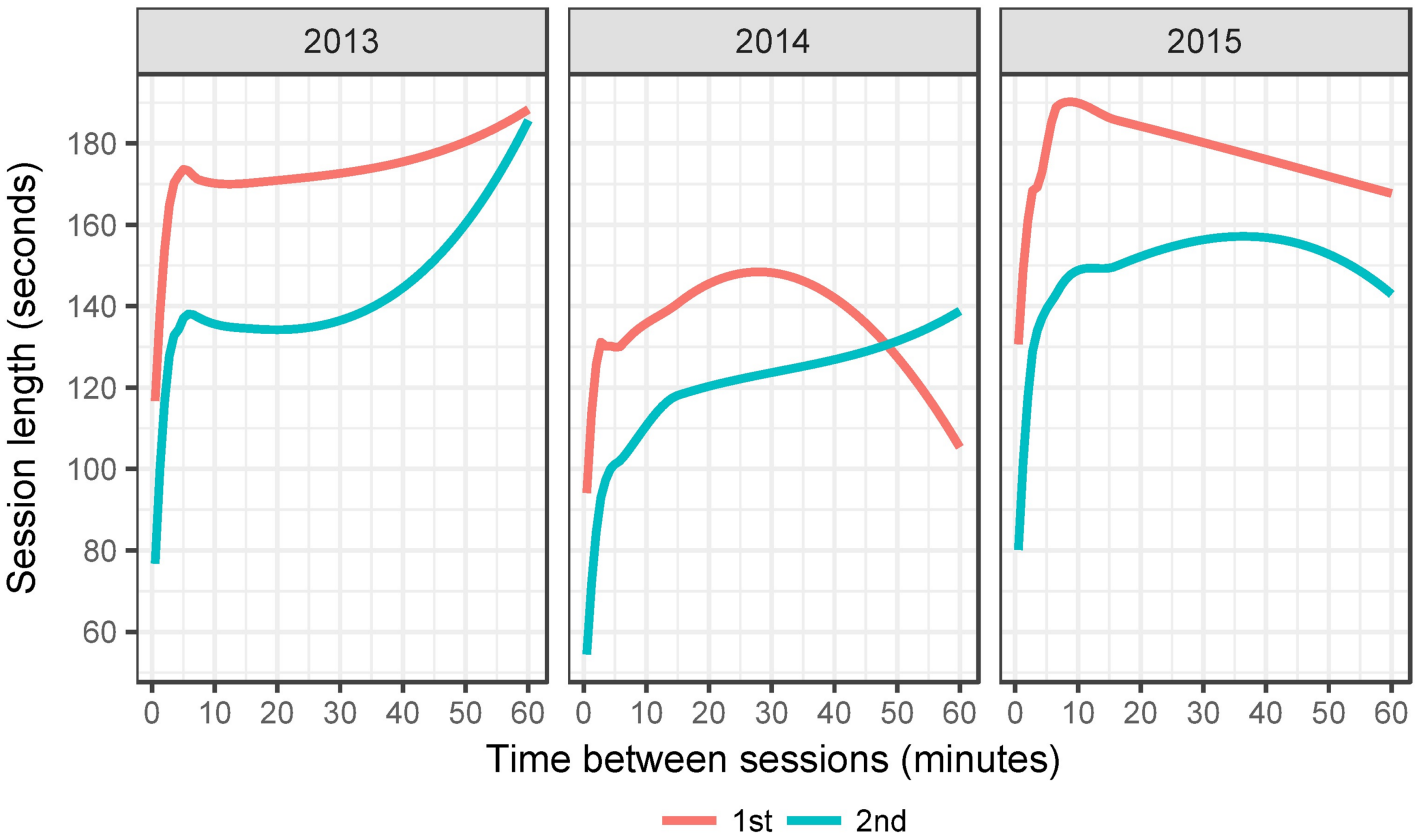
Time between sessions and average session length for paired sessions.

Before turning to examine the voting speed of the above defined pairs in detail let us examine IP-paired sessions first without applying the 10-minute time in-between and 6pm to 12pm voting or weekend voting restrictions. Fig 3 graphs the average length of both sessions against the time between them. An interesting peculiarity emerges, sessions that have less than 10 minutes in between them (i.e. the end of the first and the beginning of the second session) are clearly faster in comparison to other paired sessions and this applies to both, the first and the second session. This is already a telling detail for it would fit with a scenario when two e-voters have discussed the voting process and vote at a very fast pace, almost in unison, and it also justifies the 10 minute between sessions cutoff rule we employ for closer paired vote examination. A second remarkable aspect is that almost in all cases the second voter e-votes on average faster than the first one. Again, it suggests that the two voters have been in contact and the second one is voting faster due to the cues about the process given by the first voter. We cannot observe these scenarios directly, but the data does show persistent non-random patterns in support of that.

We will turn now to a closer examination of the e-vote pairs as defined above.

Table 3 shows the differences in average voting speeds between the two voters. The contrast is substantial, the second voter votes between 37 to 45 seconds faster than the first one and this for a process that took the first voter on average only two to three minutes. There are of course examples of pairs in the data where the second session was in fact slower than the first session, but the dominant pattern is for the second session to be faster than the first one—it holds for 65.2%, 68.5% and 64.5% of session pairs in 2013, 2014 and 2015 respectively.

**Table 3:** Average voting session length in seconds.

Year	1st voter	2nd voter	Difference
2013	162.9	126.0	37.0
2014	128.2	88.2	40.0
2015	170.2	124.7	45.5

To further demonstrate the strength of the “companion effect” on the voting speed we also examined its association with age, as prior research has shown that e-voting speed is strongly connected with age of the voter ([4], p.79). We also compared the voting speeds for lone voters and the speed of the second vote for lone voters who voted twice, given that the latter should be especially fast due to familiarity with the voting application interface and candidates.

**Fig 4:**
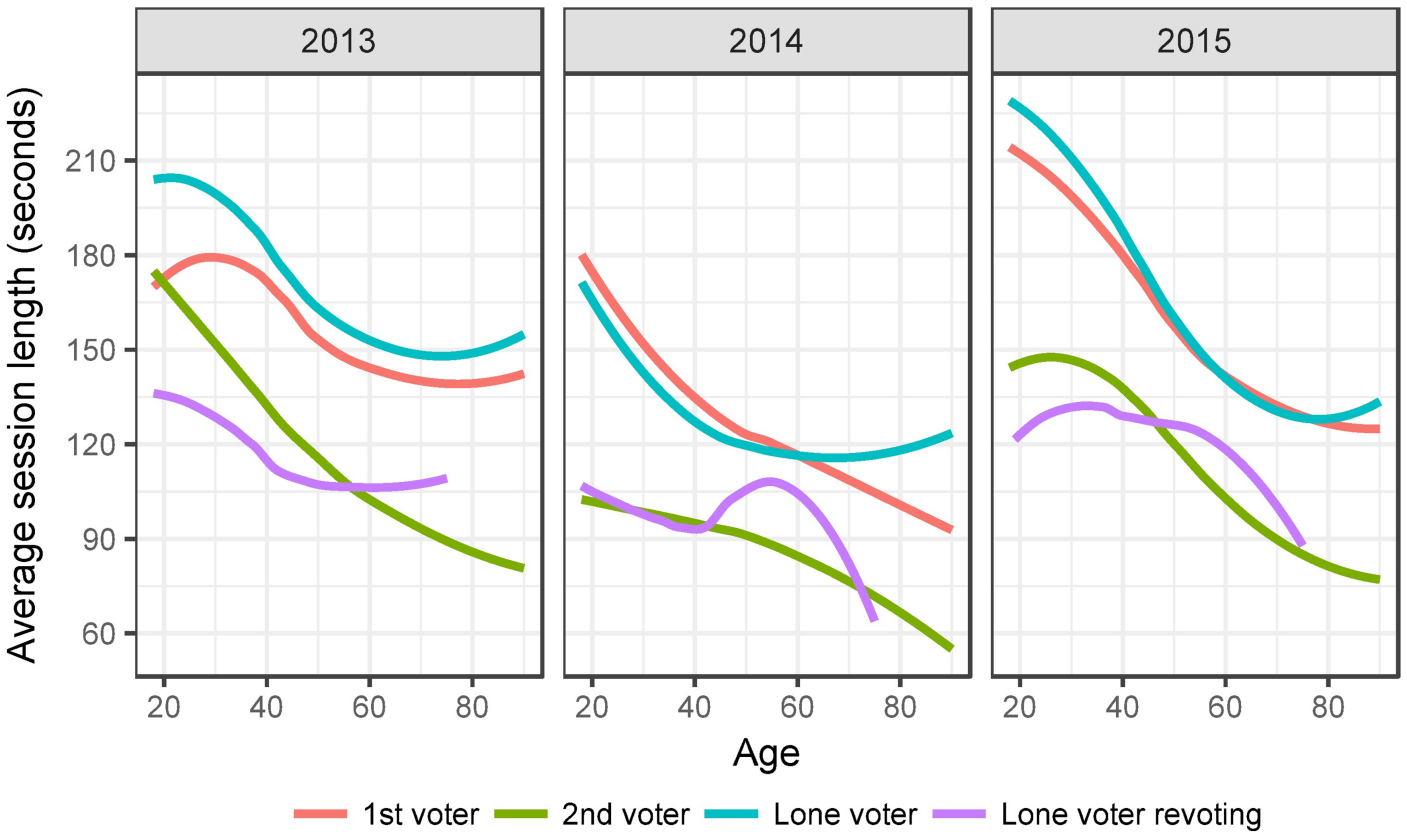
Average voting speed for voter pairs according to age.

The results are displayed in Fig 4 with three highly interesting details emerging. First, the voting speed of a lone voter is very similar to the voting speed of the first voter in a voter pair at all age levels. Second, the second voter in a pair of e-voters votes consistently faster than the first voter at all age levels. Third, and most striking, the second voter in a voter pair votes almost as fast as a lone voter who is voting for the second time. This is an especially clear indicator that the second voter takes some sort of cues from the first one, most likely by discussing the voting process or vote choice.

Table 4 gives a numerical estimation of the observed patterns. It shows the results of a linear regression where the outcome variable is the logarithm of the voting speed and input variables are whether the voter was the first or second voter in IP-paired voters, voted alone or voted alone for the second time. The reference group is the voter who voted alone. We also included the year of the election and age modeled with natural cubic splines with four degrees of freedom as controls. The first three coefficients in the table are below one, meaning the first and second voter, as well as a lone voter re-voting, all vote faster than a simple lone voter. The difference between the first voter in a voting pair and the lone voter is, however, very small, the former votes at 0.96 times the speed of the latter which translates into about 4% faster voting. The second voter in the voter pair, on the other hand, votes at 0.68 times the lone voter, meaning roughly 47% faster. Finally, the lone voter voting for a second time votes at 0.6 times the lone voter, which means 66% faster.

**Table 4:** Associations with the logarithm of e-voting speed.

	Estimate
1st voter (base: lone voter)	0.96***
2nd voter (base: lone voter)	0.68***
Lone voter re-voting (base: lone voter)	0.60***
Year 2014	0.81***
Year 2015	1.02***
Natural cubic spline of age (18–31)	0.94***
Natural cubic spline of age (32–41)	0.83***
Natural cubic spline of age (42–53)	0.83***
Natural cubic spline of age (≥ 54)	0.66***
Intercept	127.74***
N	85 989

Exponentiated OLS coefficients;

* p < 0.05,

** p < 0.01,

*** p < 0.001.

All in all, patterns seen in e-voting speed are consistent with a voting scenario where the first voter is faced with a situation similar to an individual voter voting alone who needs first to familiarize him/herself with the choices on the ballot and this takes time. The second voter is, on the other hand, most likely in some sort of contact with the first voter through observing the e-voting process or discussing the party choice and can take this as a behavioral cue resulting in a much faster voting process overall.

## Discussion

What to make of these findings? First, even with remote Internet voting the voting act clearly remains social, at least within households. The fear that Internet voting isolates the voter seems unjustified. The data exemplifies strong nonrandom patters consistent with a so called “companion effect” in voting. We saw how voters sharing an IP show systematic age patterns with either same aged male-female pairs voting together or female-female and female-male pairs with large age differences voting together—all consistent with partners or mother-daughter, mother-son and father-daughter pairs e-voting together. We also saw that e-voters sharing an IP show systematic differences in voting speed, the second vote for paired e-voters is to a considerable degree and consistently faster than the first one and has a comparable speed to when a single individual votes for a second time.

Secondly, and somewhat paradoxically, the continued social nature of the voting act in times of Internet voting puts even more responsibility on the voter to ensure the secrecy of his or her vote. Rather than a strict requirement, secrecy becomes a choice. Evidence presented here suggest a significant share of people indeed opt for the latter and use this mode of voting to engage in voting with their closest of kin. Internet voting is currently the ultimate form of remote voting. It might very well be that it goes together with a more relaxed attitude to vote secrecy. People are vocal about their voting choices in discussions and post pictures of their paper ballots on social media, Justin Timberlake got into hot water for taking a selfie in a polling booth in the latest US presidential election while the law in his home state forbids this [25]. This one prominent example is joined by thousands of other voters who do the same. Vote secrecy has become a choice and it is a sign of the times.

If this development is problematic is another question. We would argue that not really, especially given that voting research shows how voting by one family member increases the participation likelihood by others. The evidence above does suggest that Internet voting usage displays the same associations known to apply to voting since the 1950s. A positive dynamic for increased turnout might hence be realized simply by a more convenient way of sharing the fact of voting, thus spurring other people to do so and take their participation right more seriously.
